# Letter to the Editor

**DOI:** 10.1017/S0033291725100524

**Published:** 2025-06-24

**Authors:** Helerin Raikkerus

**Affiliations:** Independent researcher

**Dear Editor-in-Chief,**

Last year, an interesting review article titled ‘Discussing the concept of substance-induced psychosis (SIP)*’* by Bramness et al. was published in *Psychological Medicine.* In this paper, the authors draw attention to a variety of risk factors that can precede psychosis that appears to be substance-induced. I find it valuable that these risk factors have received attention. Nevertheless, I would like to point out that they also state that it has been proven that substance use alone is not sufficient for SIP. They base this opinion largely on clinical observations that not everyone who uses drugs becomes psychotic. However, this appears to be a negative inference from an incomplete observation and reflects a form of inferential bias (Desai, Kubo, Esserman, & Terry, 2011).

The claim that some people do not become psychotic from using substances is difficult to prove, because conducting a randomized controlled trial – where some participants would be given substances in high concentrations (i.e., amphetamine, methamphetamine, etc.) for as long as they develop or do not develop psychosis, causing deliberate harm – would be ethically questionable (Miteu, 2024). In addition, the authors themselves acknowledge:

*We fully acknowledge the capacity of some types of substances to, at times and in some, precipitate psychotic symptoms. These associations and possible mechanisms are well described by others, such as the mentioned reviews, and will not be covered here (Bramness et al., 2024).*

These sentences are difficult to understand and are contradictory to what they state in the Abstract. It has indeed been repeatedly shown that, for example, amphetamines can cause pro-psychotic changes, including interneuron death, possibly leading to disinhibited dopaminergic activity in the striatum (Hsieh, Stein, & Howells, 2014).

In addition, there seems to be an error in the abstract, because the authors claim that they have written a scoping review but have not delineated the methodology used for selecting resources in the text. Also, in the main body, it is said that it is a narrative review, which seems to be more correct. Narrative reviews, where it is not clearly stated how the articles used were chosen, can be biased by the author’s subjective opinions (Winchester & Salji, 2016). Therefore, the abstract, without reading the entire article, can mislead the reader into thinking that a more rigorous scientific approach was used to reach the incorrect conclusions mentioned.

In conclusion, the authors state as fact something that does not have sufficient scientific support. Furthermore, proving the claim that substances cannot cause psychosis by themselves is very difficult, or impossible, to verify and contradicts the main text of the article, in addition to many other scientific findings. It is misleading the reader regarding the neurobiological mechanisms of drug-induced psychosis and can be potentially detrimental to this field of research and patient care.

I recommend rewording the sentence: ‘It has been demonstrated that substance use alone is not sufficient to cause psychosis’, using less strong statements and changing the word ‘scoping’ into ‘narrative’ in the abstract.

Best Regards,

Helerin Raikkerus
